# Childhood Family Support as a Protective Factor for Adult Mental Health: Does It Hold Under Cumulative Risk?

**DOI:** 10.3390/ejihpe16060083

**Published:** 2026-06-16

**Authors:** Ateret Gewirtz-Meydan, Kimberly J. Mitchell, Jennifer E. O’Brien, Deirdre Colburn

**Affiliations:** 1 School of Social Work, Faculty of Social Welfare and Health Sciences, University of Haifa, Haifa 3103301, Israel; 2 Crimes Against Children Research Center, University of New Hampshire, Durham, NH 03824, USA; 3 School of Social Work, University of Texas at Arlington, Arlington, TX 76019, USA

**Keywords:** childhood family support, cumulative adversity, victimization, resilience, mental health distress, social determinants of health, moderation, trauma-informed care

## Abstract

Childhood family support is widely recognized as a protective factor for mental health, yet it remains unclear whether its buffering role is maintained under conditions of cumulative adversity. This study examined whether perceived childhood family support is associated with adult mental health distress and whether this association varies across levels of childhood adversity and victimization. Data were drawn from a convenience sample of 1571 adults in the United States who completed an online survey assessing childhood family support, adult mental health distress (PHQ-4), childhood victimization, non-victimization adversity, family substance use, and childhood social determinants of health (SDoH) hardship. Ordinary least squares regression models with interaction terms were used to test main and moderating effects. Greater perceived family support was associated with lower adult mental health distress (β = −0.23, *p* < 0.001). Childhood adversity (β = 0.11, *p* = 0.002) and childhood victimization (β = 0.13, *p* = 0.001) were independently associated with higher distress. Importantly, both adversity and victimization significantly moderated the association between family support and distress, such that the protective effect of family support weakened at higher levels of cumulative risk. Robustness analyses indicated that these patterns did not differ by family structure. These results suggest that while family support is beneficial, its protective effects are context-dependent and reduced under conditions of elevated risk. Overall, the findings highlight the importance of conceptualizing resilience as a multi-system process and underscore the need for interventions that address both relational and structural sources of adversity.

## 1. Introduction

Parent–child relationships represent a central developmental context shaping emotional regulation, stress responsivity, and long-term mental health. Although this construct includes multiple dimensions (e.g., attachment, perceived support, communication), the present study focuses on perceived childhood family support as a key relational resource reflecting emotional availability, encouragement, and belonging within the family context.

A substantial body of research demonstrates that perceived family support is consistently associated with better mental health outcomes across development, including lower levels of depression, anxiety, and psychological distress (Dunst, 2022; Weich et al., 2009; Wu & Lee, 2022). Importantly, perceived family support has also been conceptualized as a protective factor within broader risk frameworks (Rutter, 1987), with evidence suggesting that it may buffer against the negative effects of multiple forms of adversity, including childhood victimization and adversity (Adjei et al., 2025), and socioeconomic disadvantage reflected in social determinants of health (SDoH; Uphoff et al., 2013). At the same time, exposure to adverse childhood experiences, such as victimization, family dysfunction, and broader contextual stressors, has been robustly linked to increased risk for mental health difficulties in adulthood (McKay et al., 2022; Mehta et al., 2023; Scully et al., 2020; Wight et al., 2006).

Within this literature, growing attention has been directed toward understanding resilience processes and the conditions under which protective factors operate. Recent empirical work continues to support the protective role of perceived parental support and family connectedness, demonstrating associations with improved emotional wellbeing and reduced psychopathology among individuals exposed to adversity (Adjei et al., 2025; An et al., 2024; Hu & Cai, 2023). These protective effects are thought to operate through multiple pathways, including the development of adaptive emotion regulation capacities, the formation of secure relational schemas, and the buffering of physiological stress responses.

However, despite consistent evidence supporting its protective role, much of the existing literature has examined perceived family support in relative isolation, without fully accounting for the broader constellation of co-occurring adversities that characterize many childhood environments. Emerging evidence suggests that the protective role of family support depends on context rather than operating uniformly across individuals, and may vary not only across broader structural conditions such as socioeconomic status, but also as a function of cumulative risk, including the number, severity, and chronicity of adverse experiences (G. W. Evans et al., 2013; McLaughlin & Sheridan, 2016).

A cumulative risk framework posits that adversities rarely occur in isolation; rather, they co-occur and accumulate across domains, including victimization, non-victimization adversity, family dysfunction, and broader social and structural stressors, producing compounded effects on mental health (S. E. Evans et al., 2013; McLaughlin & Sheridan, 2016). However, rather than assuming that protective factors operate uniformly across individuals and contexts, the present study draws on a broader conceptualization of risk and resilience grounded in social work theory (Fraser et al., 1999). This perspective emphasizes that resilience emerges from the dynamic interplay between risk and protective factors, and that the effectiveness of protective processes depends on contextual conditions, including the type, severity, and accumulation of risk exposure. Within this framework, protective factors such as family support are not viewed as stable or universally effective, but rather as context-dependent resources whose impact may vary across individuals and environments. This approach allows for a more nuanced understanding of how protective processes function under conditions of elevated adversity, recognizing that the same factor may promote resilience in some contexts while offering limited protection in others.

Although social support is widely conceptualized as a protective factor that buffers the negative effects of cumulative risk on psychological well-being, contemporary resilience frameworks suggest that such buffering processes are not uniform and may depend on the broader configuration of risk and protective factors (Fraser et al., 1999). Accordingly, the effectiveness of family support may depend on the broader ecology of adversity in which it operates.

In addition, experiences of childhood victimization may further complicate the role of family support. Interpersonal forms of adversity, such as abuse or exposure to violence, are inherently relational and may co-occur within the family context itself, creating a conceptual tension in which the family may simultaneously represent both a source of support and a source of harm. Such experiences are associated with disruptions in trust, safety, and relational expectations, which may weaken the extent to which individuals are able to rely on or benefit from supportive relationships. Moreover, in contexts characterized by multiple adversities, including parental distress, substance use, family instability, and broader social disadvantage, caregivers’ capacity to provide consistent, responsive, and effective support may itself be compromised. Taken together, this suggests that the effectiveness of family support may depend not only on its presence, but also on the accumulation, type, and context of adversity in which it occurs.

Addressing these gaps is critical for both theoretical and applied reasons. From a theoretical perspective, it advances models of resilience by moving beyond assumptions of uniformly protective factors and instead emphasizing the conditional and context-dependent nature of resilience processes. From an applied perspective, it highlights the need to evaluate whether interventions that focus primarily on strengthening family relationships are sufficient for individuals exposed to high levels of adversity, or whether more comprehensive approaches that address cumulative risk and structural conditions are required.

### The Current Study

Despite extensive evidence positioning perceived family support as a protective factor, it remains unclear whether its buffering role is maintained under conditions of cumulative adversity. Prior research has often examined family support in isolation, without accounting for the co-occurrence of multiple adversities that characterize many childhood environments.

The present study addressed this gap by drawing on an integrative framework of risk and resilience (Fraser et al., 1999), which conceptualizes adaptation as emerging from the interaction between multiple risk and protective processes. While cumulative risk models emphasize the compounded impact of co-occurring adversities, this broader framework highlights that protective factors such as family support may not operate uniformly across contexts but instead vary in their effectiveness depending on the nature and accumulation of risk. Accordingly, this study examined the association between perceived childhood family support and adult mental health distress, while accounting for multiple domains of childhood risk, including victimization, non-victimization adversity, family substance use, and childhood SDoH. We further test whether the protective effect of family support varies across levels of adversity and victimization, indicating potential attenuation under higher risk. To do so, we estimated regression models assessing (a) the association between family support and mental health distress, (b) the independent contributions of cumulative risk domains, and (c) interaction effects between family support and both adversity and victimization. Finally, we examined whether these associations varied across childhood family structures. Based on prior research and resilience theory, we hypothesized that: (H1) greater childhood family support would be associated with lower adult mental health distress; (H2) higher levels of childhood adversity and victimization would be associated with greater adult distress; and (H3) the protective association between childhood family support and adult distress would be attenuated under conditions of higher adversity and victimization.

## 2. Methods

### 2.1. Participants

Data for the GrowingOp Study were collected between 24 March and 23 July 2025. Eligible participants were adults aged 18 to 60 living in the United States. A total of 3688 individuals completed the initial eligibility screener. Of these, 1444 were excluded, primarily due to age ineligibility (89.2%, n = 1289), leaving 2244 eligible participants who provided informed consent and were invited to complete the full behavioral survey.

**Figure 1:**
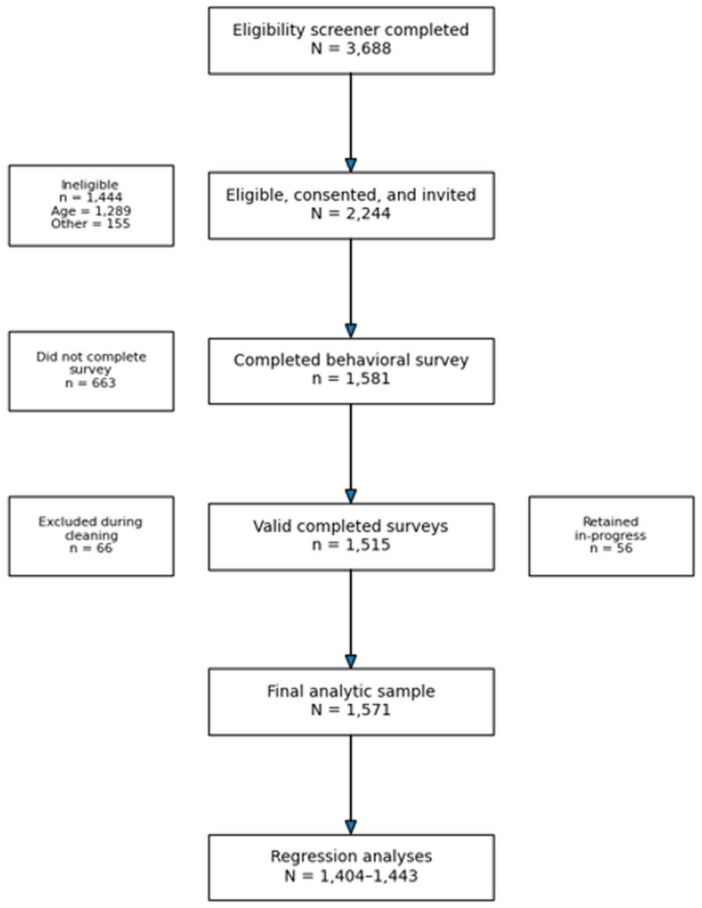
Participant flow diagram for the Growing Up with Opioids (GrowingOp) Study. Note. A total of 3688 individuals completed the eligibility screener. Of these, 1444 were ineligible, leaving 2244 eligible participants who provided informed consent and were invited to complete the behavioral survey. Following survey completion and data quality procedures, the final analytic sample consisted of 1571 participants. Regression sample sizes varied because of variable-specific missingness handled through complete-case estimation.

Of the 2244 consented participants, 1581 completed the behavioral survey, while 663 did not complete the survey. Among the completed surveys, several cases were removed during data cleaning, including those with withdrawn consent (n = 1), suspected fraudulent or duplicate responses (n = 51), ineligible age at the time of survey completion (n = 9), or unmatched screener records (n = 5). Additionally, 56 responses that were highly complete (≥92%) but initially marked as in-progress were retained and included in the final dataset. The resulting analytic sample consisted of 1571 participants. Regression analyses were conducted using complete-case estimation; therefore, analytic sample sizes varied across models because of variable-specific missingness, ranging from 1404 to 1443 participants. A participant flow diagram is provided in Figure 1. Participant characteristics are provided in Table 1.

### 2.2. Procedures

Participants were recruited nationwide through advertisements on Facebook and Instagram. Individuals who clicked on the advertisements were directed to a brief online eligibility screener. Recruitment materials used general, engagement-focused messaging (e.g., “share your experience” or “make a difference”) and did not disclose specific eligibility criteria or compensation details. Eligible individuals were invited to complete the full survey hosted on Qualtrics. Before beginning the survey, participants reviewed an informed consent document and indicated their consent electronically. Following consent, participants completed the main questionnaire. Multiple fraud detection and prevention strategies were implemented (Mitchell et al., 2025). The average survey completion time was approximately 37.5 min. Participants who completed the survey and provided a valid U.S. mailing address (excluding P.O. boxes) received a $15 Amazon gift card. Requiring a physical mailing address served as a safeguard against fraudulent participation from outside the United States, which is a known challenge in online survey research offering digital incentives (Pratt-Chapman et al., 2021). The study protocol was approved by the University of New Hampshire Institutional Review Board.

### 2.3. Measures

***Childhood family support*** was assessed using an adapted 7-item scale capturing perceived emotional support, encouragement, belongingness, and availability of guidance during childhood (before age 18). The measure was partially adapted from Turner et al. (2017) and Zimet et al. (1988). Items assess the extent to which immediate family members served as sources of strength, guidance, and tangible support. Participants were asked: “Now please tell us how true each of the following statements are about you when you were growing up (before the age of 18).” Responses were rated on a 4-point Likert scale ranging from 1 (definitely false) to 4 (definitely true). A “prefer not to answer” option was also available. One negatively worded item was reverse coded, and items were averaged to create a composite score. Scores were calculated for respondents with at least five valid responses, with higher scores indicating greater perceived family support. The adapted scale demonstrated excellent internal consistency in the current sample (Cronbach’s α = 0.88). A confirmatory factor analysis supported a unidimensional family support construct, with all standardized factor loadings statistically significant and ranging from 0.54 to 0.86.

***Childhood household structure*** was assessed using a series of items capturing the adults and caregivers with whom participants lived most of the time prior to age 18. Respondents indicated whether they lived with various caregiver types, including biological parents, step-parents, adoptive or foster caregivers, extended family members (e.g., grandparents, aunts, uncles), and non-parental adults (e.g., parental partners, friends, or other caregivers). Additional items assessed household composition and stability, including number of siblings, whether participants ever lived outside their primary caregiver household, and the number of residential moves during childhood. For descriptive purposes, these indicators were used to characterize variation in family structure (Table 1). For analytic models, a binary indicator of non-traditional family structure was created to capture exposure to caregiving arrangements beyond living exclusively with biological parents (e.g., stepfamily, extended family, foster care, or non-parental adult households). This variable was used in sensitivity analyses to examine whether associations between family support, adversity, and mental health differed by household composition.

***Childhood victimization*** was assessed using 16 items adapted from the Juvenile Victimization Questionnaire (Finkelhor et al., 2005a) and expanded to capture experiences particularly relevant to children exposed to substance misuse in the household. Additional items were developed through consultation with experts affiliated with the National Alliance for Drug Endangered Children (2025) and were designed to capture victimization experiences commonly reported in households affected by caregiver substance misuse. Items were reviewed for content relevance and consistency with existing victimization frameworks. Example items included being pressured into sexual activity in exchange for resources, exposure to individuals in the home who felt unsafe, and taking on caregiving or household duties due to caregiver impairment. Items broadly captured domains of sexual abuse, physical abuse, neglect, and exposure to violence. Respondents indicated whether each experience occurred prior to age 18 (yes/no). Affirmative responses were summed to create a cumulative victimization index, with higher scores indicating greater exposure (M = 4.12, SD = 3.69) (Finkelhor et al., 2005b, 2007, 2011). Consistent with poly-victimization research (Finkelhor et al., 2011), the measure was conceptualized as a cumulative exposure index intended to capture the breadth of victimization experiences rather than a reflective latent construct. Accordingly, items were summed to represent overall victimization burden. Because the index was designed to capture the accumulation of distinct victimization experiences rather than a single underlying latent trait, internal consistency coefficients and factor-analytic procedures were not considered appropriate evaluation methods.

***Non-victimization adversity*** was measured using 16 items assessing exposure to non-violent stressors and disruptive life events during childhood (e.g., serious illness, housing instability). Seven items were drawn from an established measure (Turner & Butler, 2003), while nine additional items were developed for the present study in consultation with field experts. The additional items were designed to capture adverse experiences commonly reported by individuals exposed to household substance misuse but not adequately represented in existing cumulative adversity measures. All items referred to experiences occurring prior to age 18 and were summed to produce a cumulative adversity score, with higher values indicating greater exposure (M = 3.79, SD = 2.70) (Turner & Butler, 2003). Consistent with cumulative stress frameworks (S. E. Evans et al., 2013; McLaughlin & Sheridan, 2016), the adversity measure was conceptualized as a formative index reflecting the accumulation of distinct adverse experiences rather than a reflective scale. Therefore, the emphasis was placed on the total number of adversities experienced rather than the internal consistency or factor structure of the component items.

***Family substance use*** during childhood was measured using a binary indicator reflecting whether respondents reported drug or alcohol problems within the household before the age of 18 (0 = no, 1 = yes). Specifically, “did anyone living in your home drink or use drugs so often that it caused problems?”

***Childhood Social Determinants of Health hardship*** was assessed using a multidimensional index capturing financial strain, food insecurity, access to preventive healthcare, neighborhood disorder, housing quality, and housing instability (Mitchell et al., 2025). Individual components included difficulty paying bills, skipping meals, lack of routine doctor and dentist visits, neighborhood problems, housing condition issues, and experiences of housing instability. For analytic models, components were standardized and averaged to create a composite index, with higher values indicating greater hardship. For descriptive purposes, an unstandardized composite was used in Table 1, and individual components were also reported to enhance interpretability. The neighborhood disorder subscale demonstrated excellent internal consistency (α = 0.91), while the housing quality subscale demonstrated acceptable internal consistency (α = 0.72).

***Adult mental health distress*** was assessed using four items from the Patient Health Questionnaire-4 (PHQ-4; Kroenke et al., 2009), which measure symptoms of anxiety and depression experienced over the past two weeks. Response options ranged from 1 (not at all) to 4 (nearly every day). For analysis, items were harmonized to a common 0–3 scale and summed to create a total distress score, with higher values indicating greater symptom burden. Scores were calculated for respondents with at least three valid items. The PHQ-4 demonstrated good internal consistency in the current sample (α = 0.86), supporting use of a summed psychological distress score.

***Demographic and socioeconomic characteristics*** were adjusted in the analyses and included age, gender identity, Hispanic/Latino ethnicity, income, educational attainment, employment status, partnership status, parental status, residential area, and adult hardship. Adult hardship was measured using a composite index capturing housing affordability, food affordability, housing insecurity, and public assistance use.

### 2.4. Data Analysis

Analyses were conducted using Stata/SE 19.5. Descriptive statistics were calculated to characterize the sample. Continuous variables are presented as means and standard deviations, and categorical variables as frequencies and percentages. In addition to reporting a composite SDoH index, individual hardship components were examined to provide greater transparency regarding the distribution of socioeconomic disadvantage. Ordinary least squares (OLS) regression models with robust standard errors were used to examine associations between childhood family support, cumulative childhood risk, and adult mental health distress. Three sets of models were estimated. Model A assessed the association between childhood family support and adult distress. Model B estimated a cumulative risk model including childhood adversity, victimization, family substance use, and SDoH hardship. Model C tested moderation by including interaction terms between family support and (a) adversity and (b) victimization. Key correlates were standardized prior to estimating interaction models to facilitate interpretation and comparability of effect sizes. Interaction effects were probed using predicted margins at low (−1 SD), mean, and high (+1 SD) levels of adversity and victimization, and visualized using margins plots. To evaluate the robustness of the findings across family contexts, additional models incorporated a binary indicator of non-traditional family structure and tested interactions between family support and family structure. Further models estimated three-way interactions between family support, adversity, and family structure, as well as between family support, victimization, and family structure. These analyses assessed whether the moderating effects of cumulative risk differed by household composition.

Missing data were handled using complete-case estimation (listwise deletion), such that participants with missing values on variables included in a given model were excluded from that analysis. Responses coded as “prefer not to answer” or “not sure” were treated as missing and were therefore excluded from model-specific analyses when relevant. Consequently, analytic sample sizes varied across models due to differences in item-level missingness, ranging from 1404 to 1443 participants.

Regression assumptions were evaluated prior to interpretation of the models. Multicollinearity was assessed using variance inflation factors (VIFs), which ranged from 1.02 to 3.24 across all models (mean VIFs = 1.42–1.61), indicating no evidence of problematic multicollinearity. Tests for heteroscedasticity suggested nonconstant residual variance; therefore, all regression models were estimated using heteroscedasticity-robust standard errors. Tests of residual normality indicated some departures from normality, although the large sample size (N > 1400) and use of robust standard errors reduce concerns regarding the validity of statistical inference. A link test indicated minor model misspecification; however, the effect was small and did not alter substantive conclusions. Independence of observations was supported by the study design, which included one observation per participant and procedures to identify and remove duplicate or fraudulent responses.

Because the study used a cross-sectional survey design, no formal a priori power analysis was conducted to determine sample size. However, a sensitivity analysis was performed using Stata’s power rsquared procedure. Using the smallest analytic sample (N = 1404), α = 0.05, and approximately 18 covariates in the full interaction models, the study had 80% power to detect an incremental R^2^ (ΔR^2^) of 0.0046 and 90% power to detect an incremental R^2^ of 0.0061 for a single focal predictor. These results indicate that the study was adequately powered to detect very small effects.

## 3. Results

### 3.1. Sample Characteristics

Table 1 presents descriptive statistics for the analytic sample (N = 1571). Participants reported moderate levels of adult mental health distress (M = 5.37, SD = 3.51). Mean childhood family support was relatively high (M = 2.96, SD = 0.77), while exposure to adversity (M = 3.89, SD = 2.86) and victimization (M = 4.12, SD = 3.69) indicated substantial cumulative childhood risk.

The unstandardized childhood SDoH hardship index had a mean of 0.77 (SD = 1.05), reflecting variability in socioeconomic and environmental disadvantage. Examination of individual components indicated that most respondents reported low levels of financial strain (83.8% rarely/never had difficulty paying bills) and food insecurity (93.8% rarely/never skipped meals). However, a notable minority experienced structural disadvantages, including lack of routine healthcare (23.3% no yearly doctor visits; 24.8% no yearly dentist visits) and housing instability (13.2%). Neighborhood disorder (M = 3.95, SD = 4.69) and housing quality problems (M = 0.91, SD = 1.47) further reflected heterogeneity in childhood environments.

The sample was predominantly cisgender women (70.9%) and non-Hispanic/Latino (92.7%), with a mean age of 33.85 years (SD = 10.06). Most participants reported at least some college education (87.6% Associate/BA+), and 75.1% were currently working. Approximately half were partnered (52.6%), and one-third had children (32.0%). Participants were distributed across rural (31.2%), suburban (39.7%), and urban (29.1%) areas.

### 3.2. Childhood Household Structure

Participants reported substantial variation in childhood household composition. While most lived with a biological mother (93.3%) and a majority with a biological father (75.4%), many experienced non-traditional family arrangements. Approximately 14.6% reported living with a stepparent, 21.3% with a grandparent, and 10.6% with extended family members such as aunts, uncles, or cousins. Nearly one in ten (9.2%) lived with non-parental adults (e.g., parental partners, friends, or other caregivers), and 22.5% reported having lived outside their primary caregiver household at some point before age 18. Residential mobility was also common, with nearly half of participants (47.2%) reporting living in three or more homes during childhood. These findings indicate considerable heterogeneity in family structure and stability across the sample. These patterns suggest that “family support” in this sample likely reflects support derived from a range of caregiving relationships, including biological, extended, and non-traditional family members, rather than exclusively from a traditional nuclear family context.

### 3.3. Associations Between Childhood Family Support and Adult Mental Health Distress

Model A (Table 2) examined the association between childhood family support and adult mental health distress. Greater family support was significantly associated with lower distress (β = −0.23, *p* < 0.001), indicating an inverse relationship independent of demographic and socioeconomic covariates. Among covariates, older age (β = −0.11, *p* = 0.001), higher education (β = −0.06, *p* = 0.018), and current employment (β = −0.07, *p* = 0.017) were associated with lower distress, while adult hardship was strongly associated with higher distress (β = 0.16, *p* < 0.001).

### 3.4. Cumulative Risk Model

Model B incorporated multiple domains of childhood adversity. Both non-victimization adversity (β = 0.11, *p* = 0.002) and victimization exposure (β = 0.13, *p* = 0.001) were significantly associated with higher adult mental health distress. In contrast, family substance use (β = 0.03, *p* = 0.293) and childhood SDoH hardship (β = −0.01, *p* = 0.780) were not independently associated with distress when considered alongside other risk domains. These findings indicate that cumulative exposure to adversity and victimization, rather than broader socioeconomic hardship alone, are key predictors of adult mental health outcomes.

### 3.5. Moderation by Childhood Adversity

Model C1 tested whether the protective association of family support varied by levels of childhood adversity. Family support remained significantly associated with lower distress (β = −0.17, *p* < 0.001), and adversity retained a positive association with distress (β = 0.13, *p* = 0.001). Importantly, the interaction between support and adversity was significant (β = 0.07, *p* = 0.011), indicating that the protective effect of family support was attenuated at higher levels of adversity.

**Figure 2:**
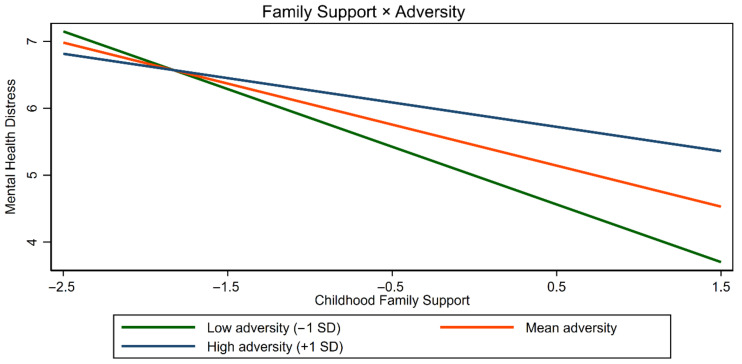
Interaction between childhood family support and cumulative childhood adversity related to adult mental health distress. Note. Predicted adult mental health distress across levels of childhood family support (standardized), shown for low (−1 SD), mean, and high (+1 SD) adversity. Higher support is associated with lower distress, but this association weakens at higher levels of adversity. Models adjust for demographic and socioeconomic covariates.

As illustrated in Figure 2, family support was associated with lower distress across all levels of adversity; however, the slope of this association was steeper among individuals with low adversity and flatter among those with high adversity. This pattern suggests that supportive family environments confer diminishing protective benefits under conditions of elevated cumulative adversity.

### 3.6. Moderation by Childhood Victimization

**Figure 3:**
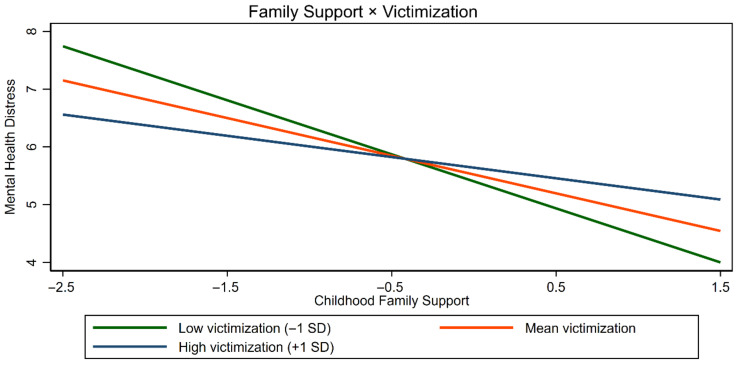
Interaction between childhood family support and childhood victimization related to adult mental health distress. Note. Predicted adult mental health distress across levels of childhood family support (standardized), shown for low (−1 SD), mean, and high (+1 SD) victimization. Higher support is associated with lower distress, but this association is attenuated at higher levels of victimization. Models adjust for demographic and socioeconomic covariates.

Model C2 examined whether childhood victimization moderated the association between family support and adult distress. Family support remained negatively associated with distress (β = −0.19, *p* < 0.001), while victimization was not independently significant in the interaction model (β = 0.03, *p* = 0.457). However, the interaction between support and victimization was significant (β = 0.08, *p* = 0.004). Figure 3 shows that the protective association of family support was strongest at low levels of victimization and weakened at higher levels. Similar to the adversity model, this pattern indicates that higher levels of victimization reduce the extent to which family support is associated with lower adult distress.

### 3.7. Summary of Findings

Across models, childhood family support was consistently associated with lower adult mental health distress. However, both cumulative adversity and victimization were associated with increased distress and significantly moderated the protective role of family support. These findings suggest that while supportive family environments are beneficial, their protective effects are not uniform and may be constrained under conditions of high cumulative risk.

### 3.8. Robustness Checks: Family Structure

To assess whether these patterns varied by family structure, additional robustness analyses were conducted (Supplemental Table S1). Models including interactions between childhood family support and a non-traditional family indicator indicated that the association between family support and adult mental health distress did not differ by household composition (b = 0.07, SE = 0.19, *p* = 0.724). Further models tested whether the moderating effects of cumulative adversity and victimization varied by family structure. The interaction between family support and adversity remained significant (b = 0.37, SE = 0.14, *p* = 0.008), as did the interaction between family support and victimization (b = 0.36, SE = 0.15, *p* = 0.015). However, three-way interaction terms were not statistically significant for either adversity (b = −0.26, SE = 0.20, *p* = 0.197) or victimization (b = −0.12, SE = 0.21, *p* = 0.576). These findings indicate that the attenuation of the protective association of childhood family support under conditions of elevated adversity and victimization does not differ by household composition.

## 4. Discussion

The present study examined whether perceived childhood family support retains its protective association with adult mental health distress when considered within a broader context of cumulative childhood adversity. Consistent with prior research, family support was associated with lower levels of distress (Fitzpatrick et al., 2024), while exposure to adversity and victimization was linked to poorer mental health outcomes (Baldwin et al., 2023; Juwariah et al., 2022). However, extending beyond these well-established patterns, the moderating impact of child victimization and adversity in the current study suggests these experiences change the relationship between family support and mental health. Specifically, the findings demonstrate that the negative association between family support and distress varies in strength and appears to weaken under conditions of elevated adversity.

This pattern is consistent with prior research suggesting that family support is beneficial, but its protective effects may be limited under conditions of severe child maltreatment (Folger & Wright, 2013; Wright & Folger, 2017). More broadly, these findings align with integrative frameworks of risk and resilience (Fraser et al., 1999), which emphasize that the impact of protective factors depends on their interaction with multiple forms of risk. Within such frameworks, adversities are understood to co-occur and correlate with compounded distress levels, thereby constraining the effectiveness of otherwise protective resources. Accordingly, even supportive relational environments may not be sufficient to fully offset the psychological burden associated with multiple and chronic stress exposures. This interpretation is also consistent with emerging work on resilience portfolios and poly-strengths, which highlights that thriving following adversity is associated with the accumulation and interaction of multiple protective factors rather than reliance on any single resource (Hamby et al., 2017, 2018).

Rather than viewing these findings as inconsistent with resilience theory, they may be better understood within a multidimensional framework of risk and protection (Fraser et al., 1999), which conceptualizes resilience as the product of interacting systems rather than the effect of a single protective factor. From this perspective, protective processes may operate through the combined and dynamic interplay of multiple resources, including self-efficacy, perceived competence, subjective health, religiosity, material resources, and social support. The impact of any single protective factor, such as family support, may therefore be limited when examined in isolation, particularly under conditions of elevated risk (Racine et al., 2020; Woodson, 2025). This interpretation underscores the importance of conceptualizing resilience as a context-dependent and systemic process, rather than as a fixed attribute or the outcome of a single buffering mechanism (S. E. Evans et al., 2013; Schury et al., 2017).

The attenuation of the protective role of family support may also reflect the relational nature of adversity itself. Experiences such as abuse and exposure to violence occur within interpersonal contexts and may be associated with disruptions in trust, perceived safety, and relationship functioning (Edwards et al., 2012; Freyd, 1997). In such cases, the family may simultaneously function as both a source of support and a source of harm, complicating the extent to which support can be effectively mobilized or internalized (Freyd, 1997). Importantly, this harm is not limited to situations in which caregivers are direct perpetrators, but may also arise in contexts of omission, such as when caregivers fail to recognize, respond to, or protect against harm inflicted by third parties, which may partially contribute to the observed attenuation of the association between family support and mental health under conditions of elevated victimization (Hatzis et al., 2017). These dynamics further highlight the importance of considering the broader relational context in which family support operates.

Another important insight from this study is that not all domains of adversity contributed equally to adult mental health distress. While victimization and non-victimization adversity were robustly associated with distress, other domains such as family substance use and childhood socioeconomic hardship were not independently associated with outcomes when considered alongside cumulative risk. This pattern aligns with prior work suggesting that interpersonal and directly experienced adversities may have more proximal and enduring effects on psychological functioning than broader structural conditions, particularly when these adversities are chronic or occur during sensitive developmental periods (Dunn et al., 2017; Vannucci et al., 2023). Another possible explanation is that the effects of childhood social determinants of health may operate indirectly through increased exposure to victimization, family instability, or other adverse experiences that were included in the models. Alternatively, once interpersonal and directly experienced adversities are considered, structural hardship alone may contribute less uniquely to adult psychological distress. These findings should not be interpreted as suggesting that childhood socioeconomic disadvantage is unimportant, but rather that its effects may be intertwined with other forms of childhood adversity. It is also possible that the relatively low prevalence of severe socioeconomic hardship in the present sample reduced variability and limited the ability to detect independent effects. At the same time, the strong association between adult hardship and mental health underscores the continued relevance of current contextual factors in shaping well-being across the life course (Vannucci et al., 2023).

### 4.1. Clinical Implications

The present findings first and foremost reinforce the importance of childhood family support as a meaningful and enduring protective factor. Even within the context of multiple adversities, higher levels of perceived family support were associated with lower levels of adult mental health distress, underscoring the lasting relevance of supportive relational environments across the life course (Deniz et al., 2025; Fitzpatrick et al., 2024; Latif et al., 2024). This highlights the continued value of interventions that aim to strengthen emotional availability, responsiveness, and a sense of belonging within families.

At the same time, the findings indicate that the protective effects of family support are not uniform and may be constrained under conditions of cumulative adversity. As such, interventions that focus solely on enhancing family relationships may be insufficient for individuals exposed to high levels of risk. Trauma-informed approaches that directly address the psychological and physiological consequences of victimization and chronic stress are likely necessary to fully support mental health outcomes in these populations. This perspective aligns with multidimensional models of resilience, which emphasize that adaptive functioning emerges from the interaction of individual, relational, and broader socioecological resources, suggesting that interventions should be trauma-informed, flexible, and tailored to individuals’ specific contexts and needs (Liu et al., 2017, 2020). In addition, it may be important to assess not only whether support is available, but whether individuals are able to mobilize and benefit from it.

From a prevention perspective, these findings suggest that reducing exposure to cumulative adversity may be critical for preserving the protective role of family support. Efforts aimed at preventing victimization, reducing family dysfunction, and addressing early-life instability may help sustain the conditions under which supportive relationships are most effective (Shonkoff et al., 2012). This includes not only reducing children’s direct exposure to harm, but also strengthening caregiver well-being and stability, thereby reinforcing the relational systems that promote resilience. Interventions that integrate trauma-informed services, economic supports, and family-centered substance use treatment may be especially well positioned to interrupt the accumulation of risk while simultaneously bolstering protective processes.

At a systems level, the results support the implementation of integrated, multi-tiered approaches that span educational, healthcare, and social service settings. Such approaches are likely to be most effective when they move beyond parallel service delivery toward coordinated, cross-sector models that align screening, referral pathways, and intervention strategies. Schools and other youth-serving institutions are particularly well positioned to function as accessible, universal platforms for identifying risk and delivering consistent, trauma-informed supports that complement family-based resources. Embedding mental health services, strengthening school–family partnerships, and integrating prevention programming within these settings may help ensure continuity of care, particularly for youth who may have limited access to formal services elsewhere (Price-Mitchell, 2011). In parallel, policies targeting structural determinants of adversity such as poverty, housing instability, and inequitable access to healthcare remain essential for addressing the upstream conditions that shape both risk exposure and the capacity of families to provide stable, supportive environments. Efforts that couple individual- and family-level interventions with broader structural supports are more likely to produce sustained improvements in developmental and behavioral outcomes (Schensul, 2009).

Finally, the consistency of the findings across family structures suggests that the observed limitations of family support are not attributable to household composition per se, but rather to the accumulation and chronicity of adversity within the caregiving context. This distinction has important implications for both research and practice, as it challenges deficit-oriented assumptions that privilege certain family forms while overlooking the conditions under which families are operating. Instead, the findings point to cumulative risk as the primary mechanism constraining the effectiveness of supportive relationships. Accordingly, clinical and policy efforts should prioritize strategies that reduce exposure to adversity, enhance caregiver capacity, and expand access to supportive resources across diverse family contexts. This includes investing in flexible, culturally responsive interventions that recognize multiple pathways to resilience and avoid narrowly prescribing a single model of family functioning (Shonkoff et al., 2012). By shifting the focus from family structure to the broader ecology of risk and protection, such approaches are better aligned with evidence indicating that resilience is shaped less by who is in the household and more by the stability, resources, and relational quality that families are able to sustain over time.

### 4.2. Limitations and Future Research Directions

This study is subject to several limitations. First, all measures relied on retrospective self-report, which may be influenced by recall bias or current mental health status. In particular, current distress may systematically color recollections of early family environments, potentially inflating associations between childhood support and adult outcomes. Second, participants were recruited through social media platforms, introducing potential self-selection bias and limiting generalizability. The sample was predominantly White and female and included a relatively large proportion of sexual minority individuals, reflecting the characteristics of those who engaged with recruitment materials and elected to participate. As such, the findings should not be interpreted as nationally representative estimates, but rather as patterns observed within this sample. In particular, the findings may have limited applicability to men, older adults, and ethnic minority groups, and future studies should employ stratified sampling approaches to better capture the diversity of childhood adversity experiences across demographic groups.

Although multiple fraud-prevention procedures were implemented, online data collection inherently carries some risk of inauthentic or duplicate responses. Third, the cross-sectional design precludes causal inference, and associations should therefore be interpreted as correlational rather than directional. An additional limitation is that cross-sectional studies cannot disentangle environmental from genetic influences; parents who provide lower support or misuse substances may also transmit psychiatric vulnerabilities genetically, and future twin or adoption study designs would be needed to isolate the true environmental contribution of childhood family support.

Additionally, psychological distress was measured using a brief four-item screener (PHQ-4) with a two-week symptom window, which may be sensitive to transient stressors unrelated to early adversity and may not capture chronic or complex trauma responses; future studies should complement such screeners with comprehensive clinical assessments or multidimensional inventories.

An additional consideration concerns the conceptualization of “family support.” Participants were not provided with a formal definition of “family,” nor were they asked to specify which relationships they referenced. However, detailed household data indicate that many participants grew up in diverse family arrangements, including stepfamilies, foster care, and households with non-parental adults. It is therefore likely that respondents interpreted “family” broadly, potentially including chosen family or non-biological caregivers. While this enhances ecological validity, it also introduces heterogeneity in the meaning of “family support” across participants. Importantly, supplemental analyses indicated that the association between family support and mental health, as well as its attenuation under higher adversity and victimization, did not differ by family structure, suggesting that this heterogeneity does not account for the primary findings.

Finally, although the study incorporated multiple domains of childhood risk, certain measurement choices, including the use of cumulative indices and standardized composites, may obscure important variability in the severity, timing, and context of specific experiences. Cumulative count indices also assume equal psychological weight across diverse adversity types, obscuring potentially important differences in severity, developmental timing, and interpersonal context. Future research would benefit from more nuanced measurement approaches, including person-centered methods or disaggregated indices, that can identify which specific adversities most strongly interact with family support and allow for the examination of causal pathways over time through longitudinal designs. Further work is needed to investigate the mechanisms underlying the attenuation of family support under conditions of high adversity, including potential roles of stress physiology, attachment processes, and individuals’ capacity to mobilize and utilize available support.

Despite these limitations, the study benefits from a large sample of U.S. adults, a comprehensive assessment of childhood environments, and the integration of cumulative risk and moderation frameworks. Together with robustness analyses across family structures, these strengths enhance confidence in the observed patterns and provide a foundation for future research examining the conditional nature of protective processes.

### 4.3. Conclusions

The findings reinforce the importance of childhood family support as a long-term protective factor for adult mental health. At the same time, the results suggest that the benefits of supportive family environments may be diminished among individuals exposed to high levels of childhood adversity and victimization. Rather than providing a uniform buffering effect, childhood support appears to offer reduced protection under conditions of cumulative childhood harm. This pattern aligns with emerging perspectives on “diminished returns” or support erosion, in which adversity correlates with reduced mental health outcomes despite prior family support. Importantly, these patterns were consistent across family structures, indicating that the diminishing protective role of family support is driven by cumulative adversity rather than differences in household composition.

## Figures and Tables

**Table 1:** Sample characteristics (N = 1571).

Variable	Mean/n	SD/%
**Key Constructs**		
Adult mental health distress	M = 5.37	SD = 3.51
Childhood family support	M = 2.96	SD = 0.77
Childhood adversity (count)	M = 3.89	SD = 2.86
Childhood victimization (count)	4.12	SD = 3.69
Childhood SDoH hardship index (raw)	M = 0.77	SD = 1.05
Hardship components		
Financial strain		
Never/rarely	1316	83.8%
Sometimes	187	11.9%
Often/always	68	4.3%
Food insecurity		
Never/rarely	1473	93.8%
Sometimes	80	5.1%
Often/always	18	1.1%
No yearly doctor visits	366	23.3%
No yearly dentist visits	389	24.8%
Neighborhood disorder	M = 3.95	SD = 4.69
Housing quality problems	M = 0.91	SD = 1.47
Any housing instability	208	13.2%
**Childhood Family Structure**		
Lived with …		
Biological mother	1465	93.3%
Biological father	1184	75.4%
Adoptive parent (any)	92	5.9%
Step-parent (any)	230	14.6%
Grandparent (any)	335	21.3%
Extended family (aunt/uncle/cousin)	167	10.6%
Non-parent adult (partner/friend/other)	144	9.2%
Foster caregiver (any)	16	1.0%
Ever lived outside primary caregiver household	353	22.5%
Number of homes before age 18		
1–2 homes	805	51.2%
3–4 homes	416	26.5%
5+ homes	326	20.7%
Number of siblings in household		
0 siblings	269	17.1%
1–2 siblings	1015	64.6%
3+ siblings	283	18.0%
**Demographics**		
Age (years)	M = 33.85	SD = 10.06
Gender identity		
Cisgender man	190	12.6%
Cisgender woman	1069	70.9%
Transgender	68	4.5%
Nonbinary	142	9.4%
Other	39	2.6%
Hispanic/Latino origin		
Not Hispanic/Latino	1440	92.7%
Hispanic/Latino	114	7.3%
Income		
Lower than average	573	37.2%
Average	651	42.3%
Higher than average	316	20.5%
Education		
<Associate/BA	195	12.4%
Associate/BA+	1376	87.6%
Employment status		
Not working	387	24.9%
Working	1169	75.1%
Partnership status		
Not partnered	742	47.4%
Married or living with a partner	822	52.6%
Parental status		
No children	1066	68.0%
Has children	502	32.0%
Area type		
Small town/rural	489	31.2%
Suburban	622	39.7%
Urban/city	457	29.1%

**Table 2:** Childhood Family Support, Cumulative Risk, and Adult Mental Health Distress.

Variables	Model A		Model B		Model C1		Model C2	
Key Constructs	β (SE)	*p* Value	β (SE)	*p* Value	β (SE)	*p* Value	β (SE)	*p* Value
Childhood family support	−0.23 (0.09)	<0.001	—		−0.17 (0.12)	<0.001	−0.19 (0.12)	<0.001
Childhood adversity	—		0.11 (0.13)	0.002	0.13 (0.13)	0.001	0.12 (0.13)	0.001
Childhood victimization	—		0.13 (0.14)	0.001	0.02 (0.16)	0.585	0.03 (0.16)	0.457
Support × Adversity	—		—		0.07 (0.10)	0.011	—	
Support × Victimization	—		—		—		0.08 (0.10)	0.004
Family substance use	—		0.03 (0.21)	0.293	0.03 (0.21)	0.289	0.03 (0.21)	0.246
Childhood SDoH hardship	—		−0.01 (0.14)	0.780	0.00 (0.14)	0.978	0.00 (0.13)	0.991
**Demographic Covariates**								
Age	−0.11 (0.01)	0.001	−0.11 (0.01)	0.001	−0.11 (0.01)	<0.001	−0.11 (0.01)	<0.001
Gender								
Cisgender man (ref)	—	—	—	—	—	—	—	—
Cisgender woman	0.05 (0.27)	0.160	0.04 (0.27)	0.258	0.04 (0.27)	0.225	0.04 (0.27)	0.206
Transgender	0.04 (0.50)	0.144	0.04 (0.49)	0.127	0.03 (0.50)	0.234	0.03 (0.50)	0.291
Nonbinary	0.08 (0.37)	0.014	0.07 (0.38)	0.035	0.06 (0.38)	0.062	0.06 (0.38)	0.071
Other	0.04 (0.63)	0.190	0.03 (0.60)	0.195	0.04 (0.60)	0.178	0.03 (0.60)	0.235
Hispanic/Latino	−0.01 (0.33)	0.763	0.00 (0.34)	0.851	−0.01 (0.33)	0.800	−0.01 (0.33)	0.780
Income								
Lower than average (ref)	—	—	—	—	—	—	—	---
Average	−0.04 (0.23)	0.253	−0.04 (0.23)	0.212	−0.04 (0.23)	0.261	−0.04 (0.23)	0.228
Higher than average	−0.06 (0.28)	0.088	−0.06 (0.28)	0.052	−0.06 (0.28)	0.082	−0.06 (0.28)	0.078
College education	−0.06 (0.29)	0.018	−0.06 (0.30)	0.037	−0.05 (0.29)	0.042	−0.05 (0.29)	0.044
Currently working	−0.07 (0.23)	0.017	−0.07 (0.24)	0.011	−0.07 (0.23)	0.016	−0.07 (0.23)	0.012
Married or living with partner	−0.03 (0.19)	0.341	−0.04 (0.19)	0.176	−0.04 (0.19)	0.193	−0.04 (0.19)	0.182
Has children	0.03 (0.24)	0.358	0.01 (0.25)	0.659	0.02 (0.24)	0.527	0.02 (0.24)	0.535
Type of community								
Rural/small town (ref)	—	—	—	—	—	—	—	---
Suburban	−0.02 (0.21)	0.512	−0.02 (0.22)	0.451	−0.02 (0.21)	0.539	−0.02 (0.21)	0.500
Urban/city	0.01 (0.23)	0.710	0.01 (0.24)	0.800	0.01 (0.24)	0.837	0.01 (0.23)	0.865
Adult hardship index	0.16 (0.52)	<0.001	0.14 (0.54)	<0.001	0.14 (0.54)	<0.001	0.14 (0.53)	<0.001
**Model Fit**								
N	1443		1407		1404		1404	
R^2^	0.16		0.16		0.18		0.19	

Note. Robust standard errors.

## Data Availability

Data is available from the corresponding author upon reasonable request.

## Supplementary Materials

The following supporting information can be downloaded at: https://www.mdpi.com/article/10.3390/ejihpe16060083/s1, Table S1: Robustness Analyses: Family Structure as Moderator of Childhood Family Support and Mental Health Distress.
