# Supplementary material for: Childhood Family Support as a Protective Factor for Adult Mental Health: Does It Hold Under Cumulative Risk?
Source: Eur J Investig Health Psychol Educ. 2026 Jun 16;16(6):83. doi: 10.3390/ejihpe16060083 (PMC13298572; doi:10.3390/ejihpe16060083)
Supplement: Supplementary file 1 [file ejihpe-16-00083-s001.zip › ejihpe-4314749-supplementary.pdf]

Supplemental Table S1. Robustness Analyses: Family Structure as Moderator of Childhood Family Support and Mental Health Distress

Variables	Model R1: Support × Family		Model R2: Support × Adversity × Family		Model R3: Support × Victimization × Family	
	β (SE)	p	β (SE)	p	β (SE)	p
**Key Constructs**						
Childhood family support	-0.65 (0.14)	< .001	-0.51 (0.15)	.001	-0.58 (0.16)	< .001
Non-traditional family	-0.06 (0.20)	.779	-0.14 (0.21)	.488	-0.17 (0.23)	.468
Support × Family	0.07 (0.19)	.724	-0.14 (0.21)	.513	-0.13 (0.25)	.612
Childhood adversity	0.46 (0.13)	.001	0.62 (0.16)	< .001	0.45 (0.13)	.001
Support × Adversity	—	—	0.37 (0.14)	.008	—	—
Adversity × Family	—	—	-0.39 (0.20)	.058	—	—
Support × Adversity × Family	—	—	-0.26 (0.20)	.197	—	—
Childhood victimization	0.05 (0.16)	.756	0.13 (0.16)	.416	0.19 (0.20)	.345
Support × Victimization	—	—	—	—	0.36 (0.15)	.015
Victimization × Family	—	—	—	—	-0.13 (0.25)	.616
Support × Victimization × Family	—	—	—	—	-0.12 (0.21)	.576
Family substance use	0.27 (0.21)	.195	0.22 (0.21)	.287	0.25 (0.21)	.237
Childhood SDoH hardship	-0.05 (0.13)	.726	0.01 (0.14)	.965	0.00 (0.14)	.981
**Model Fit**						
N	1,404		1,404		1,404	
R^2	0.18		0.19		0.19	

Notes. Standardized coefficients with robust standard errors. All models adjust for demographic and socioeconomic covariates (age, gender, ethnicity, income, education, employment, partnership status, parental status, area type, and adult hardship).
